# Multiparametric cardiovascular magnetic resonance characteristics and dynamic changes in myocardial and skeletal muscles in idiopathic inflammatory cardiomyopathy

**DOI:** 10.1186/s12968-020-00616-0

**Published:** 2020-04-09

**Authors:** Yuanwei Xu, Jianhong Sun, Ke Wan, Liuyu Yu, Jie Wang, Weihao Li, Fuyuao Yang, Jiayu Sun, Wei Cheng, David Mui, Qing Zhang, Qibing Xie, Yucheng Chen

**Affiliations:** 1 Department of Cardiology, West China Hospital, Sichuan University, Chengdu, Sichuan 610041 People’s Republic of China; 2 Department of Rheumatology and Immunology, West China Hospital, Sichuan University, Chengdu, Sichuan 610041 People’s Republic of China; 3 Department of Geriatrics, West China Hospital, Sichuan University, Chengdu, Sichuan Province China; 4 Department of Cardiology, Union Hospital, Tongji Medical College, Huazhong University of Science and Technology, Wuhan, Wuhan province China; 5 Department of Radiology, West China Hospital, Sichuan University, Chengdu, Sichuan Province China; 6 Department of Medicine, Cardiovascular Division, University of Pennsylvania, Philadelphia, USA

**Keywords:** Idiopathic inflammatory myopathies, Cardiovascular magnetic resonance, Native T1 mapping, T2 mapping, Extracellular volume, Skeletal muscles

## Abstract

**Background:**

Idiopathic inflammatory myopathy (IIM) manifest as systematic muscle involvement. Multiparametric cardiovascular magnetic resonance (CMR) could be a useful technique to detect systemic involvement and disease progression in IIM patients. This study aimed to describe the tissue characteristics and dynamic changes in myocardial and skeletal muscles after treatment in IIM patients.

**Methods:**

Forty-four consecutively recruited IIM patients (49.0 ± 12.0 years; 22 males) underwent 3 T CMR at first diagnosis, and 28 patients underwent follow-up scan after receiving standard treatment for more than 1 year. Thirty age- and sex-matched healthy subjects served as controls. The CMR protocol included: cines, T2-weighted (T2w), late gadolinium enhancement (LGE), T1 and T2 mapping, and extracellular volume (ECV) evaluated for the myocardium, and T1 and T2 mapping and ECV evaluated for skeletal muscles. Correlations between laboratory biomarkers and myocardial and skeletal tissue characteristics were analyzed. Comparisons between baseline and follow-up scans were performed using paired t-tests.

**Results:**

At baseline, IIM patients showed significantly decreased hematocrit, higher left ventricular (LV) mass index, right ventricular (RV) volume index, myocardial and skeletal native T1, T2 mapping, and ECV than healthy controls. Significant correlations were found among myocardial native T1, T2 mapping, and ECV values and N-terminal pro b-type natriuretic peptide (NT-proBNP) levels, and significant correlations between skeletal T2 mapping and inflammatory biomarkers in IIM patients.

During the follow-up, 28 patients underwent repeated CMR scan (median interval, 14.5 months, interquartile range: 13.2–15.5 months). Significant relief from clinical symptoms and decreased inflammatory biomarkers levels were observed. Significant reduction in myocardial native T1, T2, ECV, and skeletal native T1, T2, and ECV were observed during the follow-up assessment.

**Conclusions:**

Both myocardial and skeletal muscles in newly diagnosed IIM patients show distinct characteristics on multiparametric CMR. In addition, significant changes were observed in patients showing clinical remission after effective treatment, which suggests that quantitative T1, T2, and ECV techniques may have potential clinical value in IIM patients.

## Background

Idiopathic inflammatory myopathies (IIM) are a heterogeneous group of autoimmune myositis affecting skeletal muscles and multiple internal organs [1]. The typical clinical manifestations of IIM include proximal muscle weakness, myalgia, and dyspnea related to respiratory muscle dysfunction due to systemic impairment of skeletal muscles. Myocardial involvement could be subclinical, but it is a major cause of adverse outcomes in patients with IIM [1–4]. Clinical manifestations of cardiac involvement in IIM patients can range from none to arrhythmia, heart failure, and even cardiac death [5]. Previous studies have found cardiac involvement manifesting as diastolic dysfunction or decreased longitudinal strain by echocardiography [6, 7], abnormalities on electrocardiography (ECG) [6], presence of myocardial late gadolinium enhancement (LGE) [3, 8, 9], increased myocardial T1and T2 values [10–12], and increased extracellular volume (ECV) [13] by cardiovascular magnetic resonance (CMR). Although a few previous studies have reported the potential value of multiparametric CMR techniques in identifying cardiac involvement in patients with IIM [10, 11], there is still a lack of dynamic monitoring of myocardial histological changes during therapy. Meanwhile, skeletal muscle involvement is often the most severe and typical manifestation in patients with IIM. Previous studies have indicated the feasibility and potential value of evaluating thoracic skeletal tissue abnormality by using CMR [10, 12]. However, the correlation between skeletal muscle inflammation measured by mapping techniques and disease activities related to biomarkers remains uncertain.

In the present study, we hypothesized that multiparametric CMR would help identify myocardial and skeletal involvement in patients with IIM and facilitate further dynamic monitoring of the effect of therapy during the clinical convalescence period of the disease.

## Methods

### Study design and population

This was a prospective observational cohort study involving 44 patients with newly diagnosed IIM who were consecutively enrolled from January 2017 to April 2018. The diagnosis of IIM was made according to the criteria of the European Neuromuscular Centre (ENMC) International Workshop (2004) [14]. All patients were assessed by clinical symptoms, laboratory tests, electromyography (EMG) and muscle or skin biopsy. For patients with muscle weakness with or without rash, the elevated creatine kinase (CK) levels and abnormal EMG findings indicated muscle damage, and muscle biopsy was performed to confirm the diagnosis. IIM was classified as polymyositis (without rash), dermatomyositis (with rash), or autoimmune necrotizing myositis (without rash) according to specific muscular pathology. For patients without muscle weakness but rash, IIM was classified as amyopathic dermatomyositis if CK and EMG were normal but skin biopsy demonstrated reduced capillary density and deposition of the C5b-9 complement membrane attack complex on small blood-vessels along the dermal-epidermal junction. The exclusion criteria were as follows: (1) patients with other autoimmune or inflammatory diseases; (2) patients who had been diagnosed as having IIM and were treated with corticosteroids or immunosuppressive drugs; (3) patients with any known cardiovascular diseases, including coronary artery diseases, cardiomyopathy, valvular diseases, long-term poor controlled hypertension or other chronic cardiac conditions; (4) patients who were unable to tolerate CMR examination or had a contraindications to CMR; (5) patients with renal impairment defined by glomerular filtration rate < 30 ml/min/1.73m^2^; and (6) patients with incomplete or poor-quality images. For all patients with IIM, baseline image acquisition was performed before the administration of regular treatment. Thirty age- and sex-matched healthy controls without any known cardiac disease or hypertension, diabetes mellitus, cerebrovascular disease, nervous system disease, or pulmonary or renal diseases were included. The first CMR scan was performed within 1 week of the IIM diagnosis and the follow-up CMR scans were performed among patients who received at least 1 year of regular treatment.

All participants underwent the same CMR protocol and provided written informed consent. The study complied with the Declaration of Helsinki and ethical approval was obtained from the local institutional ethics committee at West China Hospital (2016355).

### CMR examination

All participants underwent CMR examinations on a 3 T scanner (MAGNETOM, Trio a Tim system; Siemens Healthineers, Erlangen, Germany) using an eight-channel phased-array body coil. All the images were acquired by breath-holding and ECG gating. A balanced steady-state free precession (bSSFP) sequence was used for cine images with continuous sampling from the basal to the apical levels on short-axis views and 2-, 3-, 4-chamber long-axis views. The typical scan parameters were as follows: repetition time (TR), 34 ms; echo time (TE), 1.3 ms; flip angle, 50°; field of view (FOV), 280 × 340 mm^2^; matrix size, 162 × 192; average temporal resolution, 35–45 ms; and slice thickness, 8 mm with no gap. Cardiac T2-weighted (T2w) images were acquired with the short TI inversion recovery (STIR) sequence on three short-axis views (basal, mid-ventricular and apical) and the 4-chamber long-axis view. The typical scan parameters were as follows: TR = 2 RR intervals during breath-hold; TE, 67 ms; flip angle, 180°; FOV, 280 × 340 mm^2^; matrix size, 166 × 256; and slice thickness, 10 mm. Phase-sensitive inversion recovery (PSIR) sequence with a segmented FLASH readout scheme was performed 10–15 min after gadolinium (Magnevist; Bayer Schering Pharma, Berlin, Germany) injection with 0.15 mmol/kg per bolus on consecutive short-axis views from the basal to apical level of LV and 2-, 3-, and 4-chamber long-axis views to obtain the LGE images. The typical scan parameters were as follows: TR, 700 ms; TE, 2.0 ms; delay time after the inversion pulse, 300–380 ms; flip angle, 20°; FOV, 260 × 340 mm^2^; matrix size, 116 × 192; and slice thickness, 8 mm. T1 mapping images were acquired on three short-axis and 4-chamber views, as in the previous sequence, by using the Modified Look-Locker Inversion-recovery (MOLLI) sequence with a 5b(3b)3b (b stands for heartbeat) scheme and a 4b(1b)3b(1b)2b scheme before and 10–15 min after the gadolinium injection. The typical scan parameters were as follows: TR, 300 ms; TE, 1.2 ms; flip angle, 35°; FOV, 270 × 320 mm^2^; matrix size, 144 × 256; and slice thickness, 8 mm. T2 mapping images were acquired by T_2_-prepared single-shot bSSFP technique before administration of gadolinium on the short- and long-axis views identical to those associated with T1 mapping images. The typical scan parameters were as follows: TR, 240 ms; TE, 1.0 ms; flip angle, 12°; FOV, 320 × 340 mm^2^; matrix, 114 × 176; slice thickness, 8 mm; and T2 preparation pulse with 0, 30, 55 ms spin echo times.

### CMR imaging analysis

#### Function and volume assessments

Biventricular volumes, ejection fraction, and LV mass were analyzed using a commercially dedicated post-processing software (Qmass, version 8.2, Medis Medical Imaging Systems, Leiden, The Netherlands) according to the standardized protocol of Society of Cardiovascular Magnetic Resonance (SCMR) post-processing guideline [15]. Biventricular volume and LV mass were indexed to the body surface area (BSA).

#### T2-weighted ratio assessment

The T2w signal intensity (SI) ratio was calculated as the ratio of the signal of the LV myocardium on mid-ventricular short-axis (SI_myo_) to the signal of the adjacent skeletal muscles (SI_sk_) on the same slice.

#### T1 and T2 mapping assessments

**Fig. 1:**
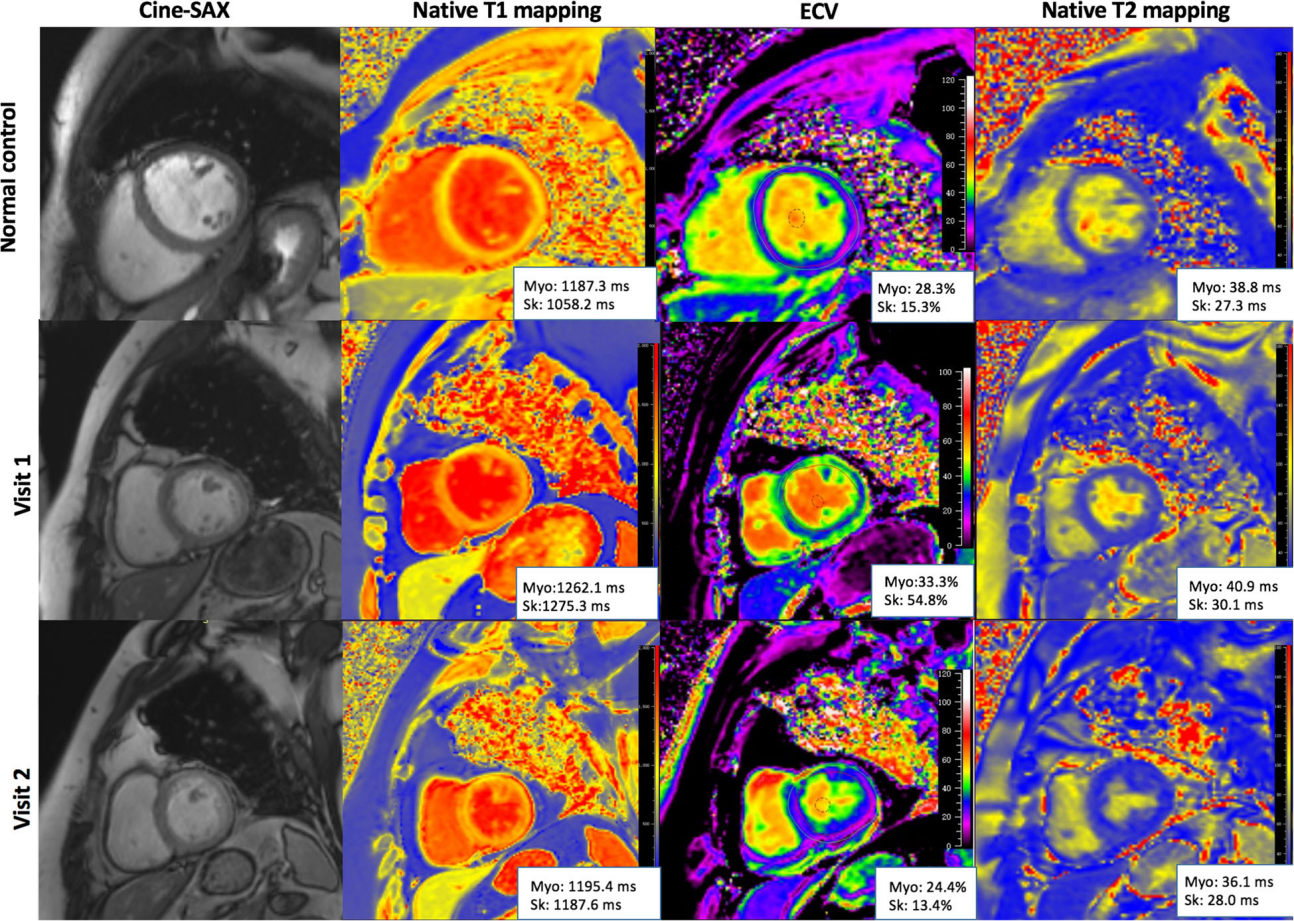
Illustrative balanced steady state free precession (bSSFP) cine, native T1, T2, and extracellular volume fraction (ECV) images on left ventricular short-axis views. Images of one healthy subject (top) and one idiopathic inflammatory myopathy (IIM) patient at baseline (visit 1) and CMR follow-up (visit 2). Abbreviation: Myo = myocardial, Ske = skeletal, SAX = short-axis; ECV = extracellular volume

Native T1, T2 relaxation times, and ECV of both the myocardium and skeletal muscles were measured on the same mid-ventricular short-axis slice by using Medis Suite 2.1 (Medis). The myocardial region of interest (ROI) was manually delineated on the end-diastolic phase with the endo- and epicardial contours to avoid endocardial trabeculations, blood pool, and epicardial fat. The selection of ROI on T1 and T2 mapping images of the adjacent skeletal muscles included five groups (pectoralis major, subscapularis, infraspinatus, upper arm, and erector spinae muscles) with carefully avoidance of adipose tissue. The analyses of tissue characteristics of skeletal muscles were performed by calculating the average values for the five groups. The native T1 and T2 relaxation times of both myocardium and skeletal muscles were generated from motion-corrected images by fitting an exponential decay curve of each pixel in ROIs. For both myocardial and skeletal muscles, the T1 relaxation time before and after contrast injection was used to calculate ECV according to the following formula: (1-hematocrit) (1/T1_muscle post_ - 1/T1_muscle pre_) / (1/T1_blood post_ - 1/T1_blood pre_). Hematocrit was obtained on the same day as the CMR examination in normal controls and within 3-days of CMR in patients. Care was taken to exclude image artifacts on myocardiaum and skeletal muscles. The illustrative images of bSSFP cine, native T1 mapping, T2 mapping, and ECV on short-axis views of a healthy subjects and one IIM patient at baseline (visit 1) and CMR follow-up (visit 2) are shown in Fig. 1.

#### LGE assessment

The presence of LGE was judged by two independent experienced CMR observers blinded to patient information. The extent of LGE was measured with the threshold defined as 3 standard derivations (SDs) above the signal of the remote normal myocardial region [15].

#### Clinical data, treatment, and follow-up

Clinical presentation and symptoms including symmetric proximal muscle weakness, rash, myalgia, and dysphagia, and laboratory biomarkers including hematocrit (Hct), C-reactive protein (CRP), erythrocyte sedimentation rate (ESR), creatine kinase (CK), creatine kinase-myocardial band (CKMB), cardiac troponins T (cTnT), and NT-proBNP were evaluated both at the time of the first diagnosis and at the follow-up within 3 days of the CMR examination in IIM patients.

After the diagnosis of IIM, corticosteroids were chosen as the first-line medications in all patients, and immunosuppressive therapies including cyclophosphamide, methotrexate, and/or mycophenolate mofetil were personalized based on the patient’s disease severity and immune response. High-dose prednisone was initially administrated with gradually tapered doses until the lowest dose which could keep the patient in sustained remission [16].

The follow-up data were last updated in Jun 2019 by collecting information by outpatient follow-up and telephone contact. Clinical remission of IIM was defined as (1) the improvement of muscle strength to normal level, accompanied by being able to move with resisting force; (2) remission of dermatitis, including the extinction of heliotrope, periorbital oedema, Gottron’s papules/sign, V-sign, shawl sign, and holster sign [14]; and (3) the normalization of laboratory tests results, including CK, ESR, and CRP levels [16].

### Statistical analysis

The normality of variables was tested using the Kolmogorov-Smirnov method. Continuous variables were expressed as mean ± SD or median (interquartile range) values. Categorical variables were expressed as percentages. Parameters between IIM patients and healthy controls were compared with independent-sample T, Mann-Whitney or chi-square tests as appropriate. The differences between baseline and follow-up parameters in the subgroup of IIM patients with follow-up CMR scans were assessed by paired t-test or Mann-Whitney U test. Logarithmic transformation was performed on biomarker parameters for normal distribution. Correlations between CMR parameters and biomarkers were evaluated using Pearson’s correlation (r). Reproducibility of tissue mapping parameters was evaluated in 20 randomly selected IIM patients. The intra- and inter-observer variability was assessed by Bland-Altman analysis (bias and 95% limit of agreement), coefficients of variation (CoVs), and intra-class correlation coefficients (ICCs). Two-tailed *p* < 0.05 was considered statistically significant. Statistical analyses were performed using MedCalc (version 11.5.1.0, Mariakerke, Belgium).

## Results

### Patient characteristics at baseline

We enrolled 46 IIM patients, among which two patients (4.3%) were excluded due to poor image quality. The study cohort consisted of 44 newly diagnosed IIM patients, and the demographic, clinical, and CMR parameters of IIM patients and healthy controls are summarized in Table 1. At baseline, IIM patients showed a significantly lower Hct, higher LV mass index and right ventricular (RV) diastolic volume index, and lower RV ejection fraction (RVEF) in comparison with age- and sex-matched controls. In the analysis of myocardial tissue characteristics, newly diagnosed IIM patients showed significantly longer native T1 (1275 ± 82 vs. 1199 ± 47 ms, *p* < 0.001) and T2 (44.2 ± 4.0 vs. 38.3 ± 2.9 ms, *p* < 0.001) relaxation times, and higher ECV (32.3% ± 6.2% vs. 26.6% ± 2.4%, *p* < 0.001) than normal controls. Eleven patients (25%) were LGE-positive with an average extent of 12.0% ± 3.1% by the threshold of 3SD. For the evaluation of skeletal tissue characteristics, IIM patients showed significantly longer native T1 (1287 ± 1499 vs. 1107 ± 70 ms, *p* < 0.001) and T2 (34.4 ± 3.1 vs. 20.6 ± 1.4 ms, *p* < 0.001) relaxation times, and higher ECV (31.1% ± 12.8% vs. 13.6% ± 3.1%, *p* < 0.001) at baseline compared to normal controls.

**Table 1:** Baseline characteristics of IIM patients compared with those of normal controls

Characteristics (units)	IIM patients (*n* = 44)	Healthy Controls (*n* = 30)	*P* value
Age (years)	49.0 ± 12.0	48.2 ± 15.6	0.232
Males, *n* (%)	22 (50.0%)	15 (50.0%)	1.000
BMI (kg/m^2^)	22.4 ± 3.0	22.8 ± 2.8	0.465
Systolic blood pressure, mmHg	122 ± 14	121 ± 11	0.673
Diastolic blood pressure, mmHg	78 ± 8	72 ± 5	0.512
Heart rate	76.5 ± 9.7	74.1 ± 7.2	0.592
Disease duration (years)	0.5 (0.2, 2.0)	–	–
**Clinical presentation**			
Proximal muscle weakness, *n* (%)	41 (93.2%)	–	–
Rash, *n* (%)	29 (65.9%)	–	–
Myalgia, *n* (%)	23 (52.3%)	–	–
Dysphagia, *n* (%)	17 (38.6%)	–	–
**Comorbidity**			
Hypertension, *n* (%)	6 (13.6%)	–	–
Diabetes, *n* (%)	1 (2.3%)	–	–
**Biomarkers**			
Hct	0.40 ± 0.05	0.43 ± 0.03	**0.003**
CRP (mg/L)	4.3 (1.7–9.7)	–	–
ESR (mm/h)	28 (23–47)	–	–
CK (U/L)	316 (79–2202)	–	–
CK-MB (ng/mL)	20.6 (5.3–54.0)	–	–
cTnT (g/L)	96 (40–269)	–	–
NT-proBNP (pg/mL)	226 (85–747)	–	–
**Medication**			
Steroids, *n* (%)	44 (100%)	–	–
Methotrexate, *n* (%)	22 (50.0%)	–	–
Cyclophosphamide, *n* (%)	12 (27.3%)	–	–
Chloroquine, *n* (%)	18 (40.9%)	–	–
Azathioprine, *n* (%)	4 (9.1%)	–	–
**Cardiac structure and function**			
LVEDVi, mL/m^2^	82.7 ± 26.6	77.1 ± 13.3	0.300
LVESVi, mL/m^2^	33.9 ± 25.1	29.1 ± 8.1	0.428
LVEF (%)	61.4 ± 11.8	62.0 ± 5.2	0.958
LVmassi (g/m^2^)	50.8 ± 15.0	43.6 ± 8.2	**0.037**
RVEDVi, mL/m^2^	80.9 ± 22.2	68.7 ± 18.5	**0.042**
RVESVi, mL/m^2^	38.2 ± 17.1	34. 3 ± 10.5	0.146
RVEF (%)	53.6 ± 9.8	57.2 ± 5.2	0.055
**Myocardial tissue characterization**			
T2w ratio	1.3 ± 0.4	1.2 ± 0.4	0.259
LGE, *n* (%)	11 (25%)	0	**–**
LGE extent, %	12.0 ± 3.1	0	**–**
Native T1 mapping-myocardial, ms	1275 ± 82	1199 ± 47	**< 0.001**
T2 mapping-myocardial, ms	44.2 ± 4.0	38.3 ± 2.9	**< 0.001**
ECV-myocardial, %	32.3 ± 6.2	26.6 ± 2.4	**< 0.001**
**Skeletal tissue characterization**			
Native T1 mapping-skeletal, ms	1287 ± 149	1107 ± 70	**< 0.001**
T2 mapping-skeletal, ms	34.4 ± 3.1	26.0 ± 1.4	**< 0.001**
ECV-skeletal, %	31.1 ± 12.8	13.6 ± 3.1	**< 0.001**

*Abbreviations*: *BMI* body mass index, *SBP* systolic blood pressure, *DBP* diastolic blood pressure, *HR* heart rate, *NYHA* New York Heart Association functional classification, *Hct* hematocrit, *CRP* C-reactive protein, *ESR* erythrocyte sedimentation rate, *CK* creatine kinase, *CK-MB* creatine kinase-MB, *TnT* Troponins T, *NT-proBNP* N-terminal pro b-type natriuretic peptide, *LVEDVi* left ventricular end-diastolic volume index, *LVESVi* left ventricular end-systolic volume index, *LVmassi* left ventricular mass index, *LVEF* left ventricular ejection fraction, *RVEDVi* right ventricular end-diastolic volume index, *RVESVi* right ventricular end-systolic volume index, *RVEF* right ventricular ejection fraction, *T2W* T2-weighted ratio, *LGE* late gadolinium enhancement, *ECV* extracellular volume fraction

Values in bold indicate *P* values < 0.05

*P* values for comparison between IIM patients and normal controls

### Correlation between tissue characteristics and cardiac structure, function and biomarkers at baseline

**Fig. 2:**
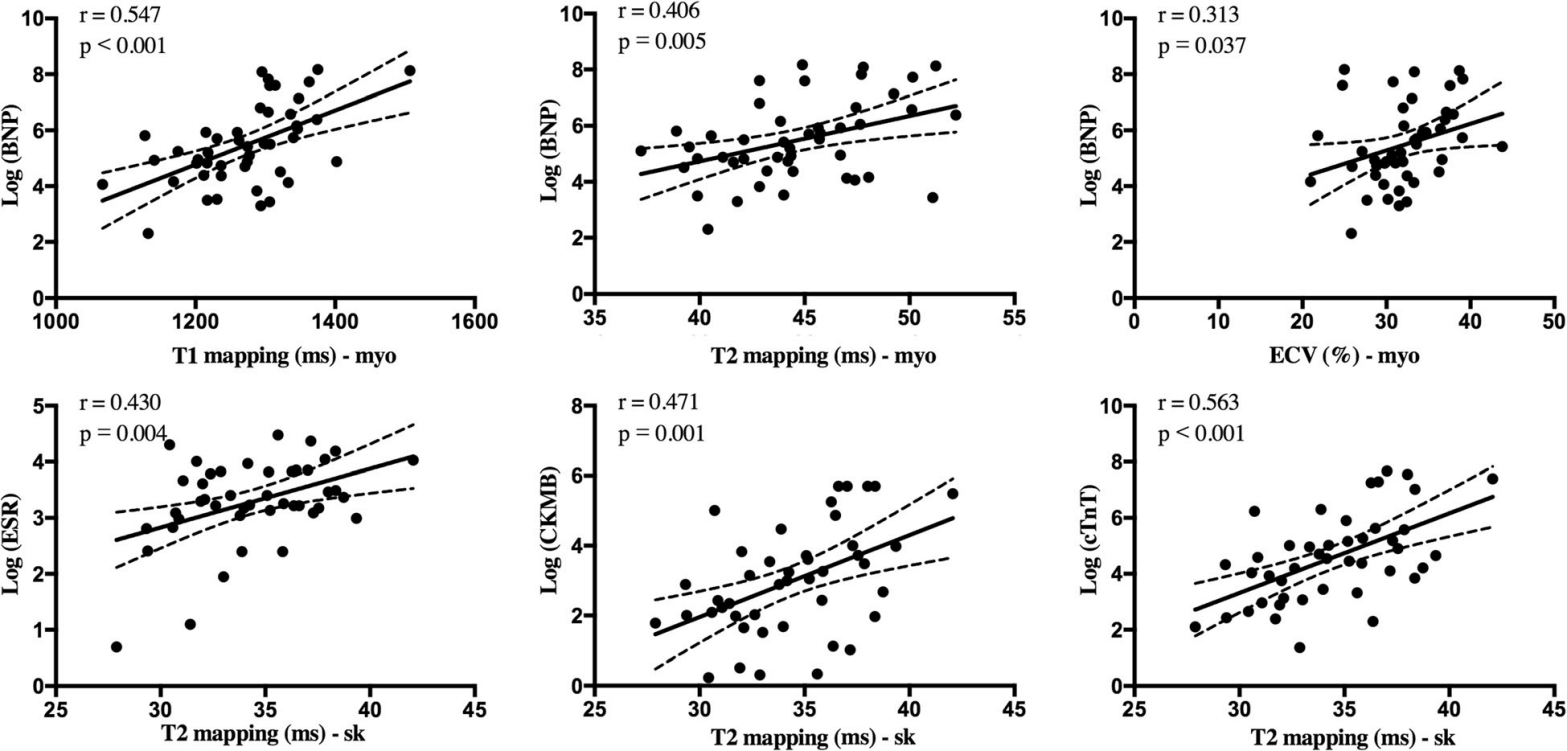
Correlation between biomarkers and quantitative myocardial and skeletal tissue characteristics at baseline

The increased myocardial native T1, T2, and ECV values were significantly correlated with a larger LV volume index, higher LV mass index, and lower LV ejection fraction (EF) Table 2. Meanwhile, elongation of the myocardial T2 relaxation time was significantly correlated with a decrease in RVEF. The myocardial native T1 (r = 0.547, *p* < 0.001), T2 (r = 0.406, *p* = 0.005), and ECV (r = 0.313, *p* = 0.037) values showed moderate positive correlation with the log NT-proBNP. Among the multiparametric tissue characteristics of skeletal muscles, we found that skeletal T2 relaxation times showed significant positive correlation with the log ESR (r = 0.430, *p* = 0.004), log CK-MB (r = 0.471, *p* = 0.001), and log cTnT (r = 0.563, *p* < 0.001) levels, while skeletal native T1 and ECV did not show significant correlations with the levels of laboratory biomarkers. The results are presented in Fig.2.

**Table 2:** Correlation between myocardial tissue characteristics with biventricular volume, ejection function, and LV mass at baseline

	LVEDVi	LVmassi	LVEF (%)	RVEDVi	RVEF (%)
native T1, ms	***r*** **= 0.392**	***r*** **= 0.358**	***r*** **= − 0.506**	*r* = 0.116	*r* = − 0.264
	***p*** **= 0.044**	***p*** **= 0.027**	***p*** **= 0.001**	*p* = 0.490	*p* = 0.110
T2 ms	***r*** **= 0.383**	***r*** **= 0.377**	***r*** **= − 0.374**	*r* = 0.278	***r*** **= − 0.340**
	***p*** **= 0.018**	***p*** **= 0.022**	***p*** **= 0.021**	*p* = 0.090	***p*** **= 0.037**
ECV, %	***r*** **= 0.310**	***r*** **= 0.329**	***r*** **= − 0.521**	*r* = 0.044	*r* = − 0.206
	***p*** **= 0.030**	***p*** **= 0.034**	***p*** **= 0.001**	*p* = 0.794	*p* = 0.215

*Abbreviations*: *LVEDVi* left ventricular end-diastolic volume index, *LVmassi* left ventricular mass index, *LVEF* left ventricular ejection fraction, *RVEDVi* right ventricular end-diastolic volume index, *RVEF* right ventricular ejection fraction

Values in bold indicates *P* values < 0.05

**Fig. 3:**
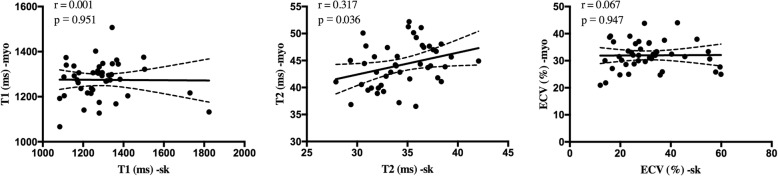
Correlation between myocardial tissue mapping characteristics and skeletal muscles at baseline

In the study, we also found that elevation of myocardial T2 value was significantly correlated with the skeletal T2 value (r = 0.317, *p* = 0.036), while no significant correlation was found among T1 or ECV values on myocardium with those on skeletal muscles. The scatterplots are presented in Fig. 3.

### Subgroup analysis of patients with follow-up CMR scans

**Fig. 4:**
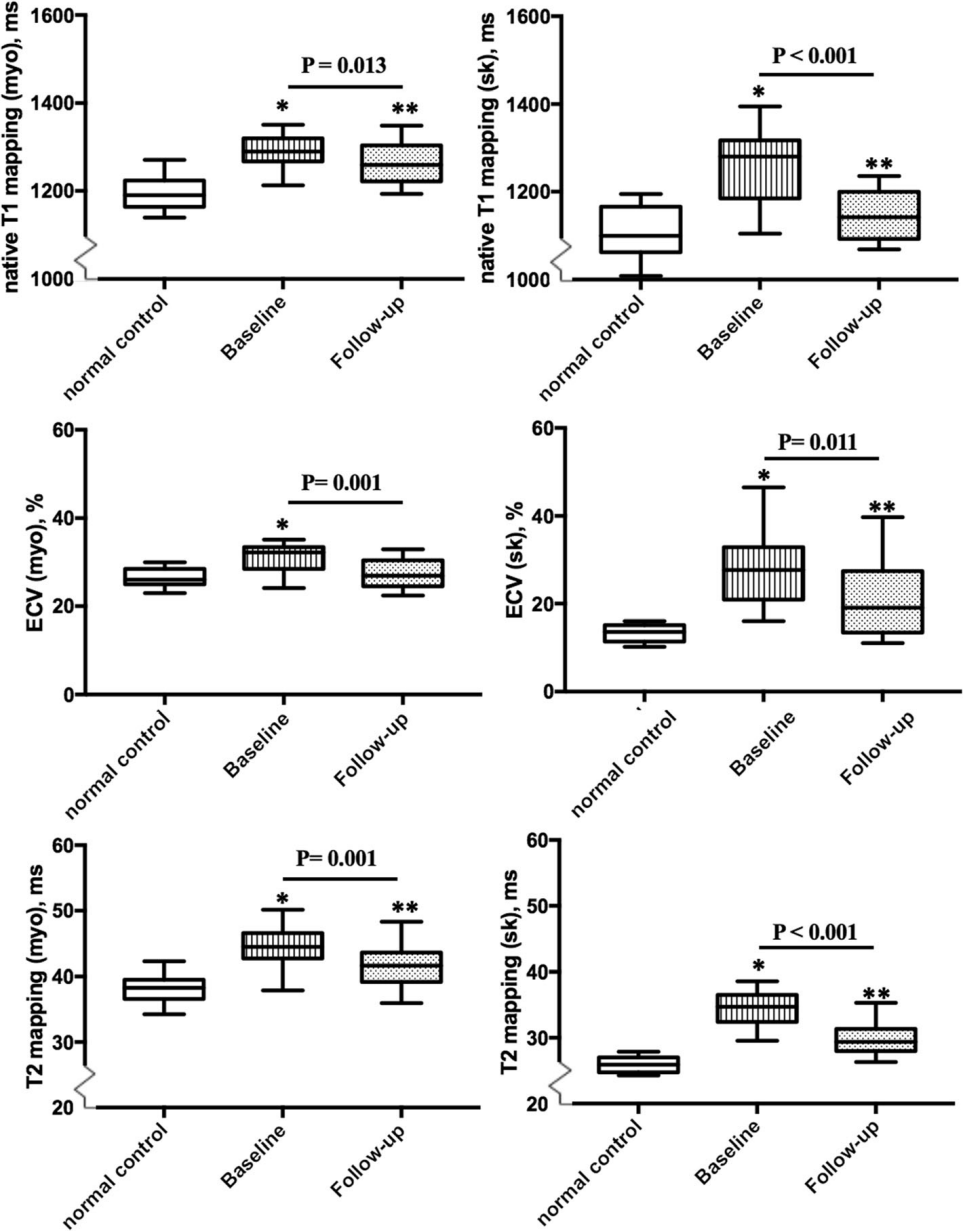
Comparison of myocardial and skeletal tissue characteristics [**a** (myocardial), **b** (skeletal): native T1 mapping; **c** (myocardial), **d** (skeletal): T2 mapping; **e** (myocardial), **f** (skeletal): ECV] among healthy controls and the IIM subgroup patients (*n* = 28) at baseline and follow-up Abbreviations: myo = myocardium; sk = skeletal muscle. The lower and upper limits of the box represent the 25th and 75th percentiles and whiskers represent the 10th to 90th range. The baseline and follow-up boxplots of IIM patients composed of 28 patients who had CMR follow-up.

During the follow-up, 28 IIM patients were arranged to consecutively undergo repeated scans with a median CMR interval of 14.5 mo (interquartile: 13.2–15.5 mo). Those who have not been treated regularly (*n* = 2), had a treatment time of less than 1 year (*n* = 6), or were reluctant to undergo a second CMR for personal reasons were excluded from the subgroup analysis. All 28 patients reached clinical remission during the CMR follow-up, with no patients demonstrating proximal muscle weakness or skin rash. Patients also showed significantly decreased CRP, CK, CK-MB, TnT and NT-proBNP levels and increased Hct during follow-up in comparision with those at baseline. While a significant decrease in LV mass index was observed (baseline: 51.2 ± 12. vs. follow-up: 45.2 ± 10.2 g/m^2^, *p* = 0.011), no significant changes were observed in the biventricular volume index or ejection function. Seven LGE-positive patients at baseline were still LGE-positive at CMR follow-up with a trend of a reduced extent of LGE with no statistically significant difference (baseline: 11.8 ± 2.9% vs. follow-up: 8.1 ± 2.0%, *p* = NS). Patients showed significantly decreased myocardial T1 mapping (baseline: 1289 ± 53 vs. follow-up: 1262 ± 59 ms, *p* = 0.013), T2 mapping (baseline: 44.4 ± 4.0 ms vs. follow-up: 41.8 ± 4.2 ms, *p* = 0.001), and ECV (baseline: 30.7 ± 4.1% vs. follow-up: 27.6 ± 4.4%, p = 0.001) during the follow-up CMR. However, the myocardial native T1 and T2 values were still significantly higher than those for normal controls. The comparison between baseline and follow-up CMR of the subgroup IIM patients is summarized in Table 3 and Fig. 4.

**Table 3:** Clinical biomarkers and CMR parameters at baseline and follow-up among IIM patients with CMR follow-up

	Baseline (*n* = 28)	Follow-up (*n* = 28)	P^*^ value
**Biomarker**			
Hct	0.40 ± 0.05	0.43 ± 0.04	**0.005**
CRP (mg/L)	5.7 (2.8–17.9)	2.2 (1.8–4.1)	**0.024**
ESR (mm/h)	26 (23–47)	22 (5–33)	0.064
CK (U/L)	479 (83–1060)	106 (75–148)	**0.038**
CK-MB (ng/mL)	24.7 (7.1–74.6)	2.5 (1.0–4.8)	**< 0.001**
cTnT (g/L)	102 (60–480)	17 (10–67)	**< 0.001**
BNP (pg/mL)	235 (72–1184)	50 (26–109)	**0.007**
**Structural and function**			
LVEDVi, ml^3^/m^2^	83.7 ± 19.4	82.3 ± 18.0	0.703
LVESVi, ml^3^/m^2^	34.0 ± 15.3	34.3 ± 13.8	0.549
LVmassi, g/m^2^	51.2 ± 12.5	45.2 ± 10.2	**0.011**
LVEF, %	59.4 ± 8.7	60.5 ± 10.5	0.395
RVEDVi, ml^3^/m^2^	81.8 ± 16.2	79.8 ± 11.6	0.279
RVESVi, ml^3^/m^2^	39.3 ± 11.8	37.4 ± 8.0	0.073
RVEF, %	53.2 ± 6.2	55.2 ± 6.8	0.492
**Myocardial tissue characteristics**			
T2w ratio	1.3 ± 0.5	1.3 ± 0.4	0.621
LGE presence, n(%)	7 (25%)	7 (25%)	1
LGE extent, %	11.8 ± 2.9	8.1 ± 2.0	0.239
Native T1-myo, ms	1289 ± 53	1262 ± 59	**0.013**
T2 mapping-myo, ms	44.4 ± 4.0	41.8 ± 4.2	**0.001**
ECV-myo, %	30.7 ± 4.1	27.6 ± 4.4	**0.001**
**Skeletal tissue characteristics**			
Native T1-sk, ms	1268 ± 103	1154 ± 73	**< 0.001**
T2 mapping-sk, ms	34.5 ± 3.5	29.7 ± 2.8	**< 0.001**
ECV-sk, %	29.2 ± 11.1	21.9 ± 11.2	**0.011**

*Abbreviations*: *Hct* hematocrit, *CRP* C-reactive protein, *ESR* erythrocyte sedimentation rate, *CK* creatine kinase, *CK-MB* creatine kinase-MB, *TnT* troponin T, *NT-proBNP* N-terminal pro b-type natriuretic peptide, *LVEDVi* left ventricular end-diastolic volume index, *LVESVi* left ventricular end-systolic volume index, *LVmassi* left ventricular mass index, *LVEF* left ventricular ejection fraction, *RVEDVi* right ventricular end-diastolic volume index, *RVESVi* right ventricular end-systolic volume index, *RVEF* right ventricular ejection fraction, *T2W* T2-weighted ratio, *LGE* late gadolinium enhancement, *ECV* extracellular volume fraction

Values in bold indicate *P* values < 0.05

P^*^ value for comparison between baseline and follow-up characteristics among IIM patients

In the assessment of skeletal tissue characteristics, patients showed significantly decreased skeletal native T1 relaxation time (baseline: 1268 ± 103 vs. baseline: 1154 ± 73 ms, *p* < 0.001), T2 relaxation time (baseline: 35.5 ± 3.5 follow-up: 29.7 ± 2.8 ms, *p* < 0.001) time, and ECV (baseline: 29.2% ± 11.1% vs. baseline: 21.9% ± 11.2%, *p* = 0.011), with the native T1, T2 and ECV values of skeletal muscles still significantly higher than those of normal controls. The results are summarized in Table 4 and presented in Fig. 4.

**Table 4:** Intra- and inter-observer reproducibility of the measurements of myocardial and skeletal mapping characteristics in IIM patients

	Bias	95% LOA	CoV (%)	ICC
**Intra-observer**				
Native T1-myo (ms)	−4.7	(− 34.5–25.1)	0.86	0.99
T2 mapping-myo (ms)	0.3	(−1.3–1.8)	1.28	0.99
ECV-myo (%)	−0.4	(− 2.2–1.3)	2.19	0.99
Native T1-sk (ms)	− 9.0	(−83.5–65.5)	2.12	0.98
T2 mapping-sk (ms)	−0.2	(− 2.6–2.1)	2.81	0.96
ECV-sk (%)	−0.1	(−2.7–2.6)	2.71	0.99
**Inter-observer**				
Native T1-myo (ms)	−5.1	(−39.2–29.0)	0.98	0.98
T2 mapping-myo (ms)	0.3	(−1.5–2.1)	1.51	0.98
ECV-myo (%)	−0.2	(−1.8–1.3)	1.80	0.98
Native T1-sk (ms)	−10.8	(−126.6–118.3)	3.52	0.93
T2 mapping-sk (ms)	−0.2	(−3.8–3.2)	4.43	0.91
ECV-sk (%)	0.3	(−2.7–3.4)	3.17	0.99

*Abbreviation*: *LOA* limit of agreement, *CoV* coefficient of variation, *ICC* intraclass correlation coefficients, *CI* confidence intervals, *ECV* extracellular volume fraction

### Inter- and inter-observer reproducibility

Table 4 shows the inter- and intra-reproducibility of the measurements obtained with native T1 mapping, T2 mapping, and ECV for myocardium and skeletal muscles. We found the reproducibility of skeletal mapping parameters (CoV ranging from 2.12 to 4.43%) to be slightly worse than those of myocardial mapping parameters (CoV ranging from 0.86 to 2.19%).

## Discussion

This study demonstrates the value of multiparametric CMR in detecting myocardial and skeletal muscles involvement in IIM patients, and dynamic changes after effective treatment. The main findings of the study are as follows: (1) multiparametric CMR could detect both myocardial and skeletal involvement in newly diagnosed IIM patients in comparision with normal controls. (2) In newly diagnosed IIM patients, the myocardial T1, T2, and ECV were correlated with LV volume, function, and the log NT-proBNP level, while skeletal T2 was significantly correlated with biomarkers reflecting disease activity. (3) The follow-up CMR showed significantly decreased myocardial T1, T2, and ECV, as well as skeletal T1, T2, and ECV, which may indicate that the involvement of myocardium and skeletal muscles was reversible. This is the first study to show the potential role of multiparametric CMR in non-invasively monitoring the treatment effect on both cardiac and skeletal systems of IIM patients.

### Myocardial and skeletal muscles involvement in IIM 

It is not uncommon for the myocardium to be affected in systemic inflammatory diseases. In this context, the clinical value of CMR parametric mapping techniques in quantifying myocardial tissue alterations has been gaining recognition in many diseases [17]. Ntusi et al. demonstrated that T1 mapping could provide added value as a biomarker for diffuse myocardial fibrosis in rheumatoid arthritis patients [18]. Biesbroek et al. found that ECV and T1 mapping were significantly associated with the disease activity in ankylosing spondylitis patients and may be a potential marker for disease monitoring [19]. In the present study, newly diagnosed IIM patients showed significantly elevated T1, T2, and ECV values in comparison with normal controls, and these findings are consistent with recent CMR studies of IIM patients [10, 12]. We also found that the myocardial native T1, T2 and ECV values were significantly correlated with the NT-proBNP level, which may suggest that.

quantitative mapping parameters can be used as potential imaging markers for detecting subclinical cardiac involvement in newly diagnosed IIM patients. The significant correlation between myocardial ECV and serum CRP level, as well as the significant correlation between myocardial and skeletal T2 values, may indicate that the degree of myocardial inflammation is positively correlated with the systematic inflammation level. In addition, for the assessment of skeletal tissue characteristics, elevated skeletal T2 relaxation time was shown to be correlated with biomarkers such as the ESR, CK, CK-MB, and cTnT levels. Our findings suggested that the skeletal inflammation reflected by CMR multiparametric mapping techniques is related to the disease activity.

### Dynamic changes in myocardial and skeletal tissue characteristics

CMR mapping technologies have shown good reproducibility and sensitivity in dynamic monitoring for different diseases. Hinojar et al. found that lupus myocarditis patients had elevated myocardial native T1 and T2 relaxation times and showed a significant decrease after intensive therapy, which suggests an attenuation of myocardial inflammation [20]. Bohnen et al. found that native myocardial T1 and T2 measurements provide excellent performance for monitoring the healing of myocarditis with a significant decline based on the stage of acute myocarditis [21]. Spieker et al. also found T2 mapping to be a good tool for monitoring myocardial inflammation in patients with suspected acute myocarditis [22]. Alkhalil et al. demonstrated that the anatomical area at risk may best be quantified by T1 mapping undertaken in the first week after reperfusion [23]. However, some previous studies have presented discrepant findings regarding the dynamic changes associated with cardiac involvement in IIM patients. Allanore et al. studied four patients and found markedly reduced LGE areas and decreased hypokinesia after 6 months of corticosteroid and immunosuppressive therapy [24]. In contrast, Mavrogeni et al. found that the signs of myocarditis were still positive on CMR reevaluation after 3 months of steroid treatment with clinical remission [25]. Péter et al. reevaluated IIM patients 3 months after starting treatment by using echocardiography and found a significant improvement in LV and RV systolic function, despite also observing deterioration of diastolic dysfunction [25]. The uncertain conclusions in previous studies may be attributable to differences in patient selection, approaches used to evaluate cardiac abnormalities, different follow-up intervals, and the insufficient sample sizes.

In the present study, we found that newly diagnosed IIM patients had significantly elevated T1, T2, and ECV values on both myocardium and skeletal muscles, which are consistent with the findings of previous studies [10, 12]. In addition, we also demonstrated for the first time that multiparametric CMR can detect dynamic changes in both myocardial and skeletal tissue characteristics after effective treatment.

Although the cellular and molecular pathophysiology underlying the myocardial involvement in IIM patients is not clear, the increased T2 relaxation time at baseline suggested that myocardial inflammation might be a major pathophysiological change in IIM patients [26]. Consistent with this speculation, we found a significant decrease in the myocardial T2 relaxation time at follow-up, which paralleled the remission of clinical symptoms and improvements in serum inflammatory biomarkers. In addition, the significant reduction in the myocardial mass index could be partially explained by the remission of myocardial inflammation. Skeletal T2 also showed a significant decrease under clinical remission. This finding suggests that IIM patients have both myocardial and skeletal inflammation at the newly diagnosed period, and that acute inflammation and edema are reversible after effective treatment. Both myocardial and skeletal T2 values served as feasible and sensitive markers for detecting myocardial and skeletal inflammation and monitoring the therapeutic effects in IIM patients. Although we found that the clinical application of these changes may be limited due to their small absolute change values, the study still provides clues for the potential application of individualized dynamic monitoring of the effect of treatment.

We found that IIM patients had elevated T1 values at both newly diagnosed and clinical remission stages in comparison with normal controls. While a previous study found that native T1 shows high diagnostic accuracy in discriminating the acute and convalescent stages of myocarditis [27], the inconsistent results of the current study indicate that the myocardial involvement in IIM patients may not only include acute inflammation but also diffuse fibrosis. The histological studies had demonstrated that the native T1 mapping relaxation time is affected by diffuse fibrosis [28, 29] and myocardial inflammation [30]. Whether the myocardial diffuse fibrosis in IIM patients is permanent or reversible over a longer term is not clear, and observational studies with a longer follow-up period are needed.

One previous study had demonstrated that in cases with coexisting inflammation and diffuse fibrosis, the ECV would reflect the sum of both pathologies [31]. ECV has shown excellent discriminative value in differentiating myocarditis patients from healthy subjects [21, 32], and declined significantly with the recovery of myocarditis [21]. In our study, myocardial ECV provided good discriminative value between newly diagnosed IIM patients and normal controls, with a significant decrease in the ECV of both the myocardium and skeletal muscle at follow-up CMR. Thus, ECV is also a promising tissue characterization marker for detecting subtle abnormalities and monitoring the recovery from inflammation of myocardial and skeletal tissue.

In contrast to the findings for quantitative T1, T2, and ECV techniques, the T2W images showed limited ability to distinguish IIM patients from normal controls. This lack of sensitivity may be explained by the concurrent abnormality on skeletal muscles [33]. The T2 mapping technique offered the advantages of quantitative measurement of T2 relaxation times without the need for skeletal muscles as reference. Our finding suggests that T2W images have limited value for evaluation of IIM patients, consistent with the findings of a previous study [26].

In the current study, IIM patients with positive LGE at baseline still showed LGE during follow-up, without a significant decrease in the LGE extent. This may be explained by the fact that LGE reflects irreversible myocardial injury [34]. Previous studies have indicated that the presence of LGE may reflect chronic and active myocardial inflammation [34, 35].

### Correlations between tissue characterization parameters and cardiac function and structure

In the current study, we found that elevated myocardial native T1, T2, and ECV significantly correlated with LV systolic dysfunction and enlargement, while the myocardial T2 value also correlated with RV dysfunction. These results suggest that myocardial inflammation and the possible presence of myocardial fibrosis in IIM patients can adversely impact cardiac function and structure.

### Study limitations

This study had several limitations. First, this is a single-center study with a relatively small number of patients. Further studies with larger sample sizes and external validation are needed. Second, not all IIM patients enrolled at baseline underwent follow-up CMR scans. Although this could potentially lead to study bias, we focused on the subsequent observation CMR scans to analyze the dynamic changes in both myocardial and skeletal tissue characterizations. Third, the imaging data for the myocardium were not supported by biopsy findings. Future studies on the prognostic value of multiparametric CMR in IIM patients are required to validate these findings.

## Conclusions

The study demonstrates that multiparametric CMR could allow early detection of myocardial and skeletal muscles involvement in IIM patients, and help monitor dynamic changes after effective treatment.

## Data Availability

The datasets acquired and/or analyzed during the current study are available from the corresponding author on reasonable request.

## Abbreviations

BSA: Body surface area
bSSFP: Balanced steady-state free precession
CK: Creatine kinase
CK-MB: Creatine kinase-myocardial band
CMR: Cardiovascular magnetic resonance
CoV: Coefficients of variation
CRP: C-reaction protein
cTnT: Cardiac troponins T
ECG Electrocardiogram ECV: Extracellular volume fraction
EMG: Electromyogram
ENMC: European Neuromuscular Centre
ESR: Erythrocyte sedimentation rate
FOV: Field of view
HCT: Hematocrit
HF: Heart failure
ICC: Intra-class correlation coefficient
IIM: Idiopathic inflammatory myopathies
LGE: Late gadolinium enhancement
LV: Left ventricle/left ventricular
LVEF: Left ventricular ejection fraction
MOLLI: Modified Look-Locker inversion recovery
NT-proBNP: N-terminal pro b-type natriuretic peptide
PSIR: Phase sensitive inversion recovery
ROI: Region of interest
RV: Right ventricle/right ventricular
RVEF: Right ventricular ejection fraction
SCMR: Society of Cardiovascular Magnetic Resonance
SD: Standard derivation
SI: Signal intensity
SI_myo_: Signal intensity of myocardium
SI_sk_: Signal intensity of skeletal muscle
STIR: Short-tau inversion recovery
T2w: T2-wighted
TE: Echo time
TR: Repetition time
